# Olmesartan-induced Enteropathy: A Rare Side Effect of a Common Medication

**DOI:** 10.7759/cureus.6400

**Published:** 2019-12-17

**Authors:** Mustafa Sher, Michael Murray, Lloyd McGuire, Sonya Fitzpatrick, Jagadeesh Kurtkoti

**Affiliations:** 1 Neurological Surgery, Griffith University, Southport, AUS; 2 Internal Medicine: Gastroenterology, Pindara Specialist Suites - Pindara Private Hospital, Benowa, AUS; 3 Pathology, Helix Pathology, Southport, AUS; 4 Immunology, Basis Pathology, Brisbane, AUS; 5 Internal Medicine: Nephrology, Griffith University, Southport, AUS

**Keywords:** olmesartan, enteropathy

## Abstract

Ischemic heart disease and stroke are the leading causes of mortality worldwide according to the World Health Organization. Hypertension is a major factor in the development of these diseases. Olmesartan is an angiotensin II receptor blocker (ARB) indicated in the treatment of hypertension. There are several case reports describing sprue-like enteropathy caused by olmesartan. We report on a 72-year-old patient referred to our hospital for work-up of chronic diarrhoea, vomiting and weight-loss, and villous atrophy on intestinal biopsy. The patient’s symptoms abated upon cessation of olmesartan. This case illustrates the need for a thorough medication history and regular review during work-up. We hope it will add to the current understanding of this rare phenomenon.

## Introduction

Hypertension is a contributing factor to the development of a number of chronic diseases, such as cardiovascular disease, stroke, chronic kidney disease, retinopathy, and dementia. Reduction of blood pressure by anti-hypertensive treatment reduces cardiovascular morbidity and mortality [1].

Olmesartan is an oral angiotensin II receptor blocker (ARB) approved for use in the treatment of hypertension by the Therapeutic Goods Administration (TGA) since 2005 and available on the Pharmaceutical Benefits Scheme (PBS) [1]. Olmesartan blocks the binding of angiotensin II to the AT1 receptor on vascular smooth muscle [1]. By doing so it blocks the vasoconstrictor effect of angiotensin II without potentiating bradykinin activity, unlike angiotensin-converting-enzyme inhibitors (ACEIs) which are less selective.

There have been few case reports, and some case series, reporting olmesartan-induced enteropathy (OIE) and this has now been acknowledged by the TGA [1]. Imperatore and other co-workers (2016) reported a similar case in Italy of a 60-year-old man presenting with chronic, nonbloody diarrhea, nausea, anorexia, and consequent weight-loss [2]. The patient’s past medical history included hypertension that was being treated with a combination of olmesartan medoxomil and amlodipine (40 mg + 10 mg daily, respectively) for the last three years, benign prostatic hyperplasia, and two sessile colonic polyps removed by diathermic loop excision [2]. Empirical therapies failed to resolve the patient’s illness. He had negative serology for coeliac disease despite carrying the HLA-DQ2 haplotype and six months of gluten free diet (GFD) did not resolve his symptoms [2]. At admission to the Department of Gastroenterology at the University of Naples Federico II (Italy) the patient had raised erythrocyte sedimentation rate, C-reactive protein and fecal calprotectin [2], suggesting intestinal inflammation and duodenal villous atrophy. Other investigation results being negative, budesonide 9 mg/day was trialled ex-adjuvantibus, giving “good but short-lived” resolution of diarrhoea [2]. Upon reviewing the literature, the treating team eventually became aware of case reports of OIE and ceased olmesartan therapy [2]. Clinical and endoscopic resolution of symptoms followed and the patient was stable at six month follow-up; his hypertension being treated by an ACEI [2].

We report on a 72-year-old patient with a noncoeliac sprue-like enteropathy that was only resolved by ceasing olmesartan therapy. Our case is unique in that the patient’s symptoms relapsed when olmesartan was re-commenced during work-up, and our patient had simultaneous low-grade colitis and recent history of uveitis.

## Case presentation

The patient was a 72-year-old lady with a clinical history of hypertension treated with olmesartan (Olmetec) and metoprolol for many years. She also suffered from hypothyroidism and was on thyroxine (100 mcg). She presented in June 2016 with six weeks' history of profuse watery diarrhoea three to four times a day in the context of one month of vomiting and eight kilograms weight loss. She felt weak and had difficulty walking upstairs. She was given a prescription for loperamide by her general practitioner (GP) without effect.

The patient had a history of ovarian cancer that was treated by total hysterectomy and bilateral salpingo-oophorectomy. She was seen a year prior to this presentation and had no evidence of recurrence. She had experienced a recent episode of uveitis treated with topical steroids.

Physical examination was unremarkable. She was stable, afebrile, and alert. She had a soft, nontender abdomen and bowel sounds were present. Lungs were clear on auscultation and there were no adventitious sounds. Cardiovascular system was unremarkable.

Investigations

**Figure 1:**
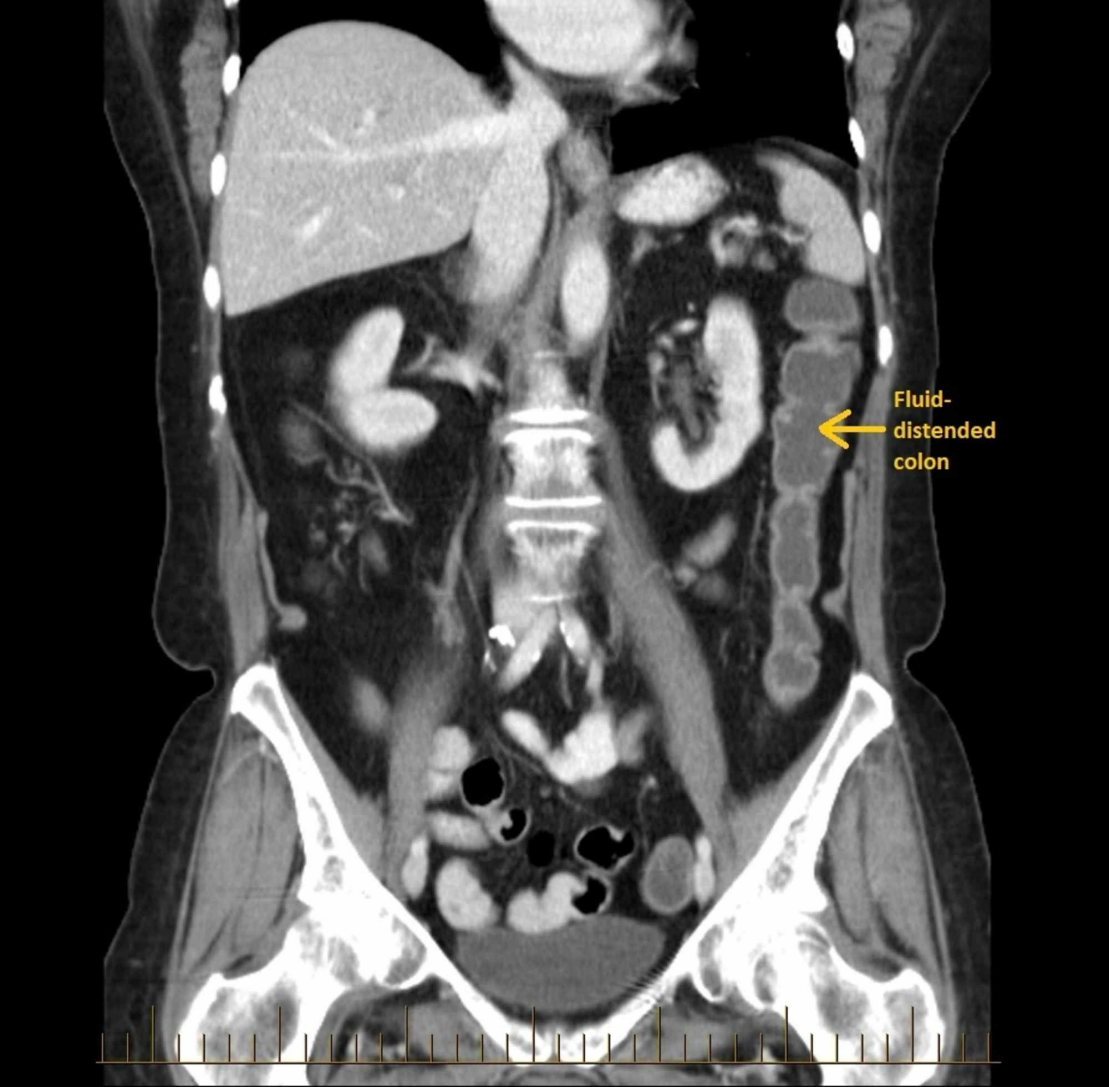
Abdominal CT scan whilst symptomatic on olmesartan showed generalized borderline fluid-filled distension of the colon without thickening or concerning focal lesion of the bowel and no adjacent fat stranding or perforation.

The CT scan showed generalized borderline fluid-filled distension of the colon without thickening or concerning focal lesion of the bowel and no definite adjacent fat stranding or perforation (Figure 1). There were no findings of diverticulitis or hepatitis. There was cholelithiasis without features of cholecystitis.

Olmesartan and metoprolol were ceased, and the patient was observed closely.

Bacterial fecal polymerase chain reaction (PCR) and screening for Clostridium difficile were negative. Calprotectin 1200 and neutrophil elastase were not detected. Adenovirus was detected by PCR, however, did not explain the chronicity of the patient’s symptoms. Potassium was 4.7 mmol/L, estimated glomerular filtration rate was 56 mL/min, and creatinine 90 µmol/L.

The patient’s symptoms improved. The patient was discharged for follow-up in a gastroenterology clinic.

Endoscopic ultrasonography in mid-2016 revealed normal pancreas, ducts, liver, and common bile duct.

**Figure 2:**
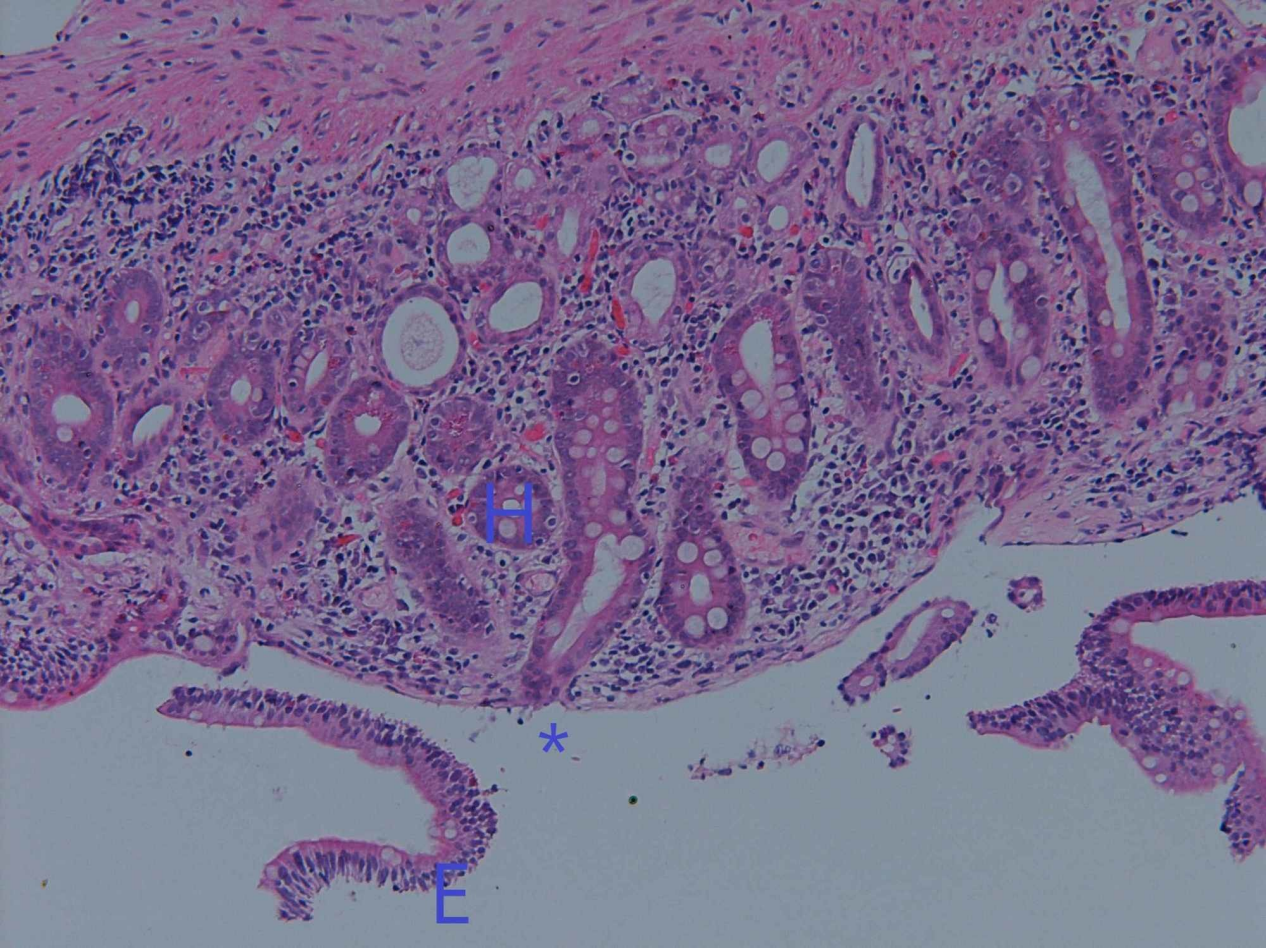
Duodenal biopsy from 2016 showing total diffuse villous atrophy (*) and crypt hyperplasia (H), as well as some loss of surface epithelium (E). The crypt has intraepithelial infiltration of lymphocytes.

Duodenal biopsy showed total diffuse villous atrophy and crypt hyperplasia (Figure 2). There was some loss of surface epithelium, and patchy basement membrane thickening was noted including some clumped collagen. The morphology was not typical of collagenous enteritis. No parasites were seen. The crypts had intra-epithelial infiltration of lymphocytes. Serology testing for coeliac disease was negative with anti-tissue transglutaminase IgA less than two units per milliliter (RR<20).

**Figure 3:**
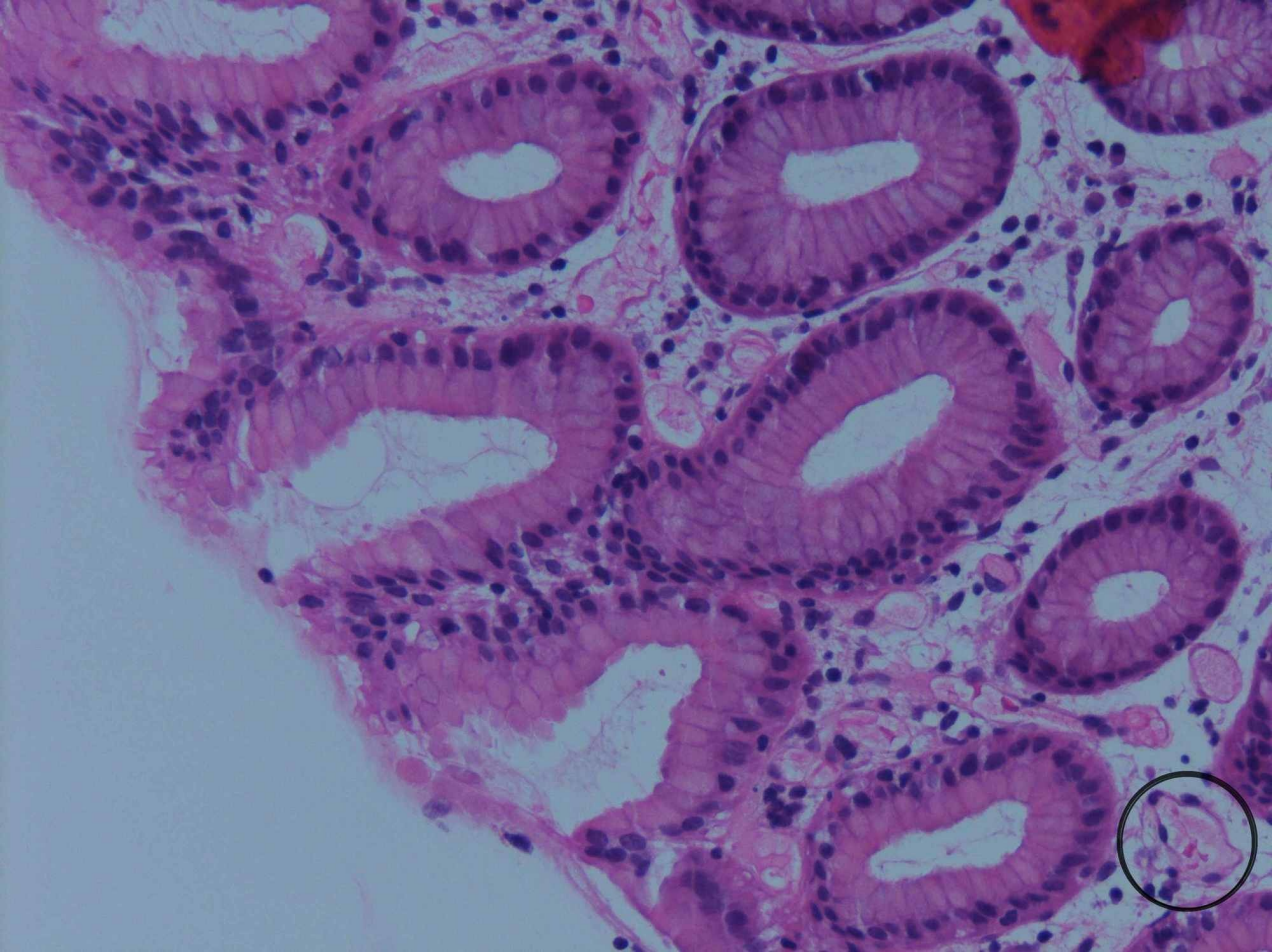
Random biopsy of the colon showing minimal nonspecific architectural changes, mild chronic inflammation, and vascular ectasia in the lamina propria (circled).

A random biopsy of the colon (Figure 3) showed minimal nonspecific architectural changes, mild chronic inflammation, and vascular ectasia in the lamina propria (circled). Low grade colitis has been noted in OIE.

**Figure 4:**
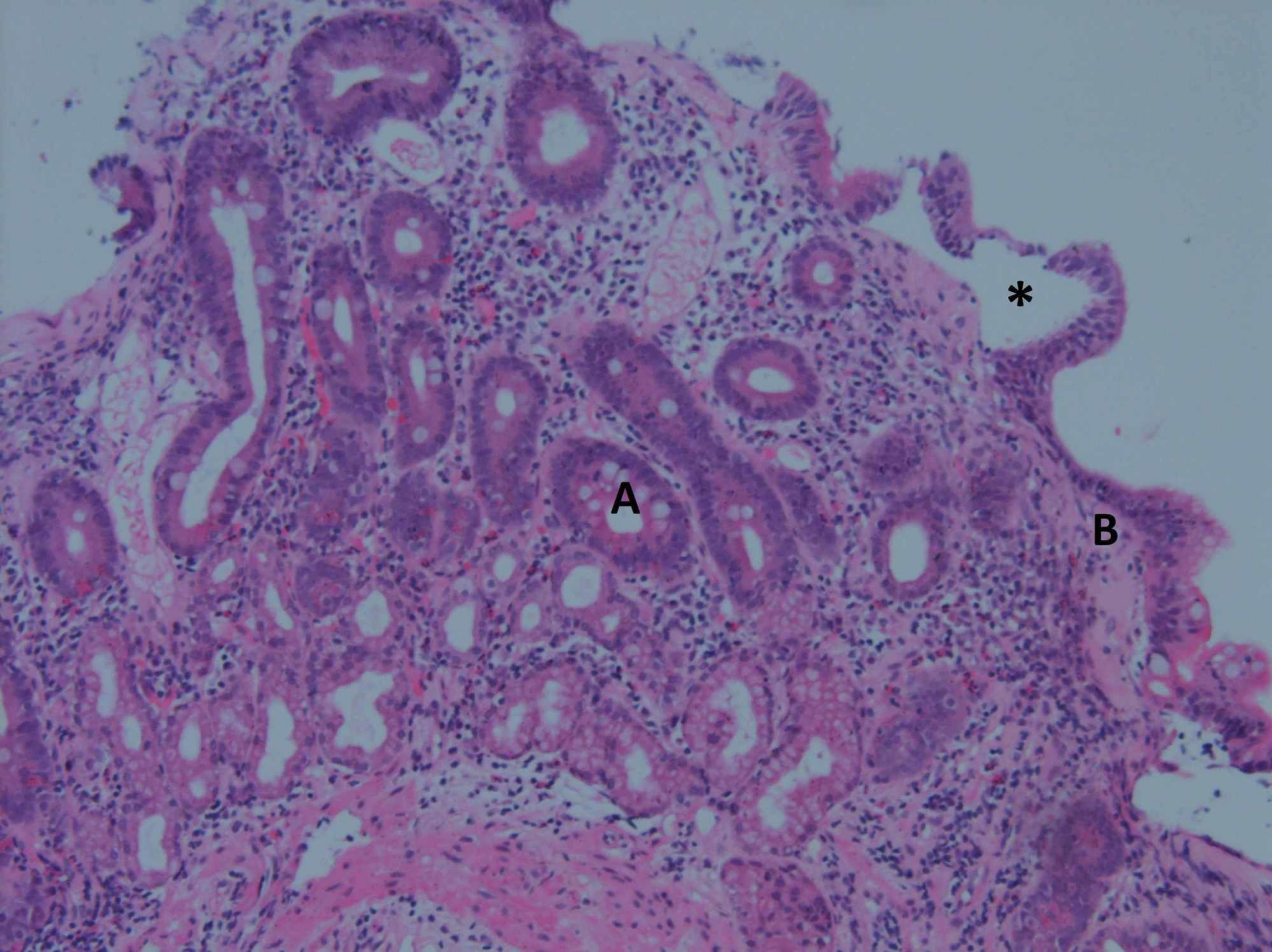
Repeat duodenal biopsy after second presentation in 2017 showing villous atrophy (*), associated with basement membrane thickening (B) and some apoptosis in crypts (A).

The patient presented again to her GP in March 2017 with a blood pressure of 170/100 and olmesartan and metoprolol were restarted. She was otherwise well at this point. One month later she presented to the Gold Coast University Hospital (GCUH) with diarrhoea, vomiting, and weight-loss of nine kilograms. Repeat biopsy showed villous atrophy associated with basement membrane thickening and some apoptosis in the crypts (Figure 4). Olmesartan was ceased due to low BP and the patient began to improve after this point.

Treatment and outcome

It was decided that olmesartan was the probable cause of the patient’s enteropathy, and it was withdrawn from her medication plan. The patient was instead put on Lercanidipine, a calcium channel blocker, for management of her hypertension.

The patient made a symptomatic recovery after cessation of olmesartan. A follow-up colonoscopy was not conducted.

## Discussion

Rubio-Tapia et al. were the first to report OIE [3]. They reported a case series of 22 patients presenting with chronic diarrhoea and weight-loss, with 15 patients (68%) having villous atrophy and mucosal inflammation on biopsy [3]. Symptoms began months to years after commencement of olmesartan therapy [3]. There was absence of tissue transglutaminase antibodies and a gluten-free diet was ineffectual; patients only recovered after discontinuation of olmesartan [3].

Whilst some case reports have described isolated small bowel effects from olmesartan, few authors have reported colonic involvement. Among those, Gallivan and Brown (2014) reported sub-epithelial basement membrane thickening, lymphocytic invasion into the epithelial lining of the colon, chronic inflammation with eosinophil infiltration into the lamina propria, and apoptotic bodies within the colonic crypts [4].

The pathophysiology of OIE is not fully understood. The possibility of our patient’s uveitis being associated with immunologic disease is a consideration, particularly as it is hypothesized that there is a predisposition to OIE in patients with an autoimmune background [5]. Further studies on ARB-associated enteropathy and its pathophysiology is warranted. 

## Conclusions

Olmesartan is a rare cause of enteropathy and should be considered in the differential diagnosis of noncoeliac sprue-like illness. This case illustrates the importance of taking and reviewing a thorough medication history during work-up for gastrointestinal conditions, and the importance of keeping an open mind about uncommon side effects of commonly used medications.
